# Quantifying mechanical forces during vertebrate morphogenesis

**DOI:** 10.1038/s41563-024-01942-9

**Published:** 2024-07-05

**Authors:** Eirini Maniou, Silvia Todros, Anna Urciuolo, Dale A. Moulding, Michael Magnussen, Ioakeim Ampartzidis, Luca Brandolino, Pietro Bellet, Monica Giomo, Piero G. Pavan, Gabriel L. Galea, Nicola Elvassore

**Affiliations:** 1 Developmental Biology and Cancer, UCL GOS Institute of Child Health, London, UK; 2 Department of Industrial Engineering, University of Padua, Padua, Italy; 3 Veneto Institute of Molecular Medicine, Padua, Italy; 4 Istituto di Ricerca Pediatrica, Fondazione Città della Speranza, Padua, Italy; 5 Department of Molecular Medicine, University of Padua, Padua, Italy

**Keywords:** Tissues, Biopolymers in vivo

## Abstract

Morphogenesis requires embryonic cells to generate forces and perform mechanical work to shape their tissues. Incorrect functioning of these force fields can lead to congenital malformations. Understanding these dynamic processes requires the quantification and profiling of three-dimensional mechanics during evolving vertebrate morphogenesis. Here we describe elastic spring-like force sensors with micrometre-level resolution, fabricated by intravital three-dimensional bioprinting directly in the closing neural tubes of growing chicken embryos. Integration of calibrated sensor read-outs with computational mechanical modelling allows direct quantification of the forces and work performed by the embryonic tissues. As they displace towards the embryonic midline, the two halves of the closing neural tube reach a compression of over a hundred nano-newtons during neural fold apposition. Pharmacological inhibition of Rho-associated kinase to decrease the pro-closure force shows the existence of active anti-closure forces, which progressively widen the neural tube and must be overcome to achieve neural tube closure. Overall, our approach and findings highlight the intricate interplay between mechanical forces and tissue morphogenesis.

## Abstract (web summary)

Hydrogel force sensors directly bioprinted into embryonic tissues quantify the forces driving tissue remodelling and reveal the existence of mechanisms that counteract tissue morphogenesis.

## Main

Morphogenesis is the quintessentially biomechanical process by which embryonic cells change their tissue’s shape, establishing the form necessary for subsequent organ function. Intricate dynamics between multiscale force fields and biochemical factors acting on heterogeneous cell populations impose evolving geometrical constraints and allow developing embryos to robustly self-organize organ rudiments^1,2^. Failure of morphogenesis and uncoupling between ‘passive’ mechanical properties and ‘active’ force generation, associated with genetic and environmental factors, produce congenital anomalies that remain a major cause of infant mortality globally. Across Europe, nearly 27 out of every 1,000 births are affected by a congenital anomaly, and 33% of affected children do not survive infancy^3^. Neural tube defects (NTDs) remain among the most common and severe congenital malformations^4^. These defects are caused by failure to close the embryonic neural tube, a biophysical process that has long served as a clinically relevant paradigm of morphogenesis^5^.

The neural tube is the embryonic precursor of the vertebrate central nervous system. It mechanically closes through dorsal bending and medial apposition of the initially flat neuroepithelium into a continuous tube. Closure is a complex, coordinated, dynamic process in which active multiscale cell-generated forces exceed residual tissue tensions to produce tubular morphogenesis^6–9^. Genetic or teratogenic disruptions of neural tube closure biomechanics can cause NTDs^10,11^. Although essential, the analysis of morphogenetic forces generated during neural tube closure has not yet been tractable. Step-changing biomechanical technologies have invariably produced novel insights into the mechanics of life. At subcellular levels, Förster resonance energy transfer (FRET)-based tensile strain sensors have revealed differential cell cortical tension, highest in the apically constricting neuroepithelium in *Xenopus*^12^. Spring-like force sensors that can be implanted into large cells have revealed intracellular forces generated during cell shape changes^13^. Approaches that quantify mechanical pressure or tensile stresses have also provided unique insights into cell compaction or shear stress in embryos^14–16^. However, none of these methods quantify dynamic tissue-level forces. Classical force-measuring experiments using ferromagnetic ‘dumbbells’ revealed peak neurulation forces in the nano-newton range in two amphibian species^17^. Destructive testing of *Xenopus* tissue explants has quantified >5 µN of force against millimetre-scale confinements^18^. Tissue-level cantilever probing also allows partial quantification of tissue mechanics limited to narrow force directions and/or tissue landscapes^19–22^. Difficulties in combining cantilever force measurements with high-resolution microscopy to ensure correct tissue contact, tissue slippage or sheer under the cantilever tip, and challenges of calibrating individual delicate cantilevers partially immersed in aqueous medium, all limit the widespread use of such cantilevers.

We envisioned designing a simple, versatile and widely applicable force sensor technology to quantify the embryonic tissue-level biomechanics of morphogenesis, providing a temporal profile of evolving forces and mechanical work generated in living vertebrate embryos. This aim imposes predetermined requirements. The force sensor technology must be compatible with embryo development to provide mechanical read-outs over developmentally relevant time frames of several hours, not seconds to minutes. Physical force sensors must have compliant elastic properties sensitive to the imposition of forces in the nano-newton to micro-newton range. Achieving elastic compliance requires control over sensor shape and the bulk and chemical properties. Sensor size, spatial position and orientation should be precisely controllable through in situ microfabrication at cell-level and tissue-level length scales during live imaging. These structural properties must be flexible and adaptable to circumvent inter-embryonic variability, providing a generalizable solution that does not require prior knowledge of individual embryo morphology or tissue mechanical properties.

Here we describe the development of a force quantification method applicable to vertebrate morphogenesis by creating spring-like nano-newton force sensors within living chicken embryos by means of an intravital three-dimensional (i3D) bioprinting approach. This i3D bioprinting has previously been used to print millimetre-scale structures under mouse epidermis of the skin, dura mater of the brain and epimysium of the skeletal muscle in vivo ^23^. We redeveloped this technique to enable micrometre-scale photo-crosslinking of biocompatible photo-active polymers in a three-dimensional (3D) elastic hydrogel. Combined with live-imaging microscopy, i3D-bioprinted spring-like force sensors allow dynamic quantification of neurulation mechanics. As proof of principle, we demonstrate quantifiable disruption of closure mechanics in Rho-associated protein kinase (ROCK)-inhibited embryos.

## Adaptable bioprinting of elastic shapes in embryos

**Fig. 1:**
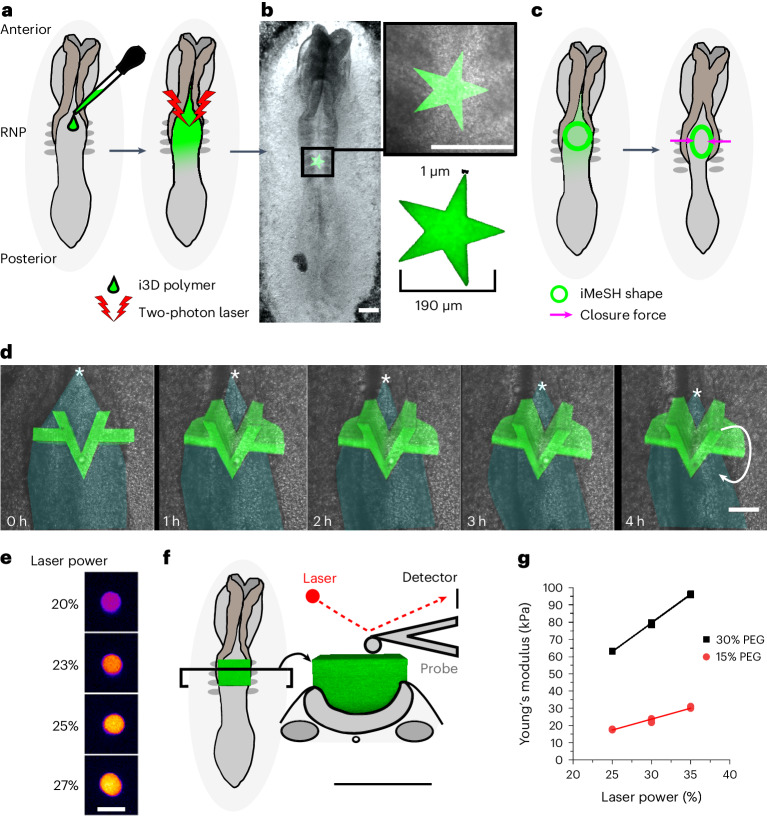
The i3D bioprinting with accurately determined position, geometry and stiffness. **a**, Schematic of a chicken embryo illustrating the experimental workflow: 2–3 µl i3D polymer is pipetted directly onto the rhombocervical neuropore (RNP) and photo-crosslinked with a two-photon laser. The iMeSH structures are shown in green throughout. **b**, Stereoscope image of an embryo with a star shape photo- crosslinked on the flat neural plate. Scale bars, 200 µm. The star dimensions are indicated in the inset. **c**, Schematic showing iMeSH compression by apposition of the neural folds. **d**, Time-lapse images showing the sequential displacement of a rigid iMeSH shape, shown as a 3D confocal reconstruction superimposed on the embryo imaged with transmitted light. Cyan shading, open neural tube; *, zippering point; arrow indicates rotation of the printed shape; scale bar, 50 µm. The times are shown. **e**, Fire lookup table showing the autofluorescence of iMeSH photo-crosslinked with the indicated laser powers on the same embryo. Scale bar, 25 µm. **f**, Schematic illustration of AFM stiffness testing of an iMeSH shape; 3D reconstructions of the shape are shown superimposed on a dorsal and transverse schematic of the embryo. Scale bar, 100 µm. **g**, AFM quantification of iMeSH crosslinked on an embryo at the indicated laser powers. The values were calculated from AFM indentations performed at a rate of 0.5 μm s^–1^ and depths of 1 μm (30% 7-hydroxycoumarin-3-carboxylic acid (HCC) polyethylene glycol (PEG)) or 2 μm (15% PEG). Source data

**Extended Data Fig. 1:**
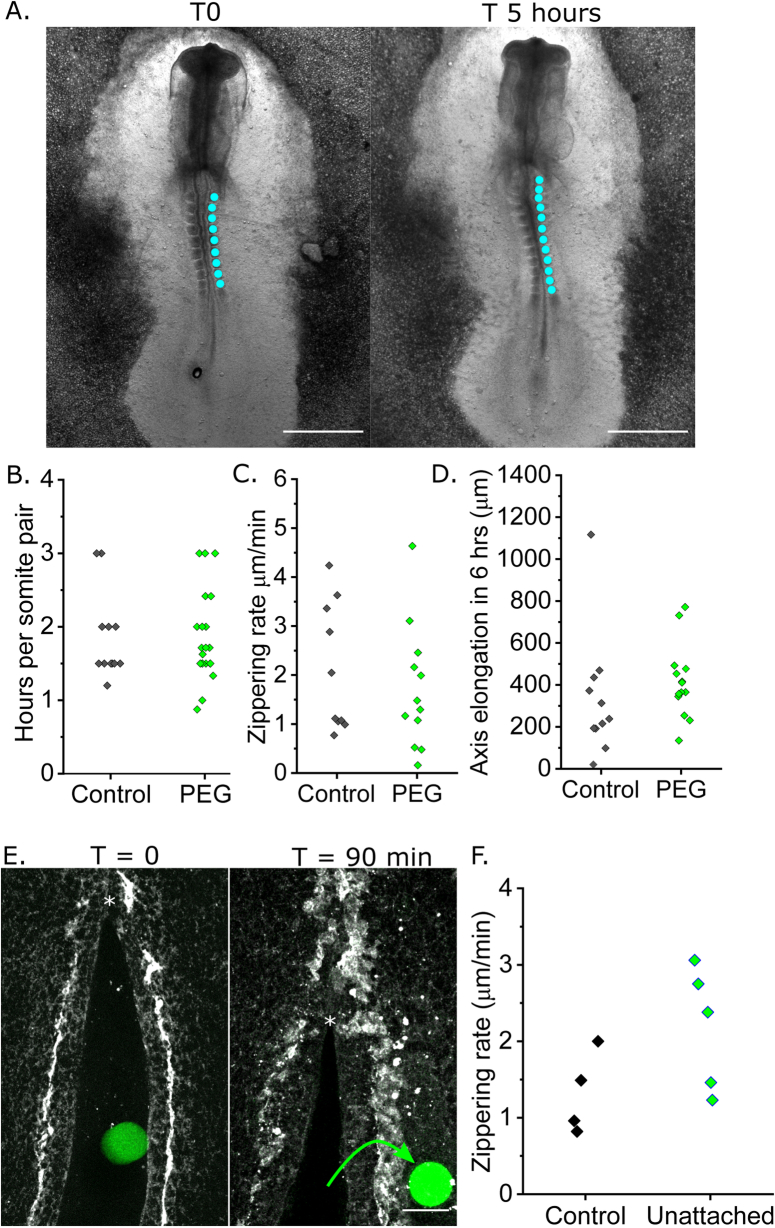
i3D polymer and printing do not diminish embryo development. **a**. Brightfield images of a chick embryo in EC culture at T0 and T 5 hours. Cyan dots indicate somites. Scale = 500 μm. **b-d**. Quantification of parameters to compare growth between control embryos in EC culture and those treated with 30% HCC PEG liquid polymer. Points represent independent embryos. B. Rate of somite gain, n = 13 (control) and n = 21 (PEG). C. Caudal zippering point progression relative to a somite landmark, n = 10 (control) and n = 12 (PEG). D. Embryo axis elongation, n = 12 (control) and n = 15 (PEG). **e**. Sequential confocal images of a chick embryo posterior neuropore soon after i3D printing a pillar between its neural folds but not attached to its tissues, and 90 minutes later when theNunattached cylinder had been extruded from the neuropore (green arrow). * indicates thezippering point, scale bar = 50 μm. **f**. Quantification of rate of zippering point progression in the posterior neuropores of control embryos live-imaged without i3D printing (N = 4) and in embryos with unattached pillars printed inside their neuropore lumen (N = 5). Points represent individual embryos. Source data

We present a highly tractable procedure to generate elastic shapes directly in living embryos, adapted to tissue geometry, without requiring specialist equipment beyond readily available two-photon microscopes (Fig. 1a). Two-photon i3D bioprinting enables the creation of 3D shapes with high positional and structural accuracy directly in confocal-imaged chick embryos (Fig. 1a,b). Optimized experimental conditions reproducibly produce predesigned structures in anatomically defined regions of interest, namely the closing neural tube (Fig. 1b). A star shape is shown to illustrate the versatility of the printing dimensions over two orders of magnitude, from ~1 µm at the tips to a nearly 200 µm inter-tip span in the example shown (Fig. 1b). Alternative crosslinked geometries can readily be defined and adapted to the closing neural tubes of individual embryos (Fig. 1b–d). Neither the liquid polymer nor crosslinking of unattached structures impacts embryo development (Extended Data Fig. 1).

Closure of the neural tube requires medial apposition of the neural folds, physically narrowing the open region and allowing the progression of dorsal midline fusion by the ‘zippering point’^5,24^ (schematically illustrated in Fig. 1c). The neural tube zippering speed is not significantly affected by i3D printing (Extended Data Fig. 1e,f). As zippering advances and embryos continue to develop, rigid i3D-printed structures can be displaced and ejected from the closing tube (Fig. 1d and Extended Data Fig. 1e). Their displacement indicates the generation of mechanical forces by the embryo. To quantify these forces, we adapted i3D bioprinting to create elastic, compliant shapes anchored to the closing neural folds, such that their deformation serves as a read-out of forces generated by medial apposition of the neural folds (Fig. 1c). We will refer to these structures as intravital mechano-sensory hydrogels (iMeSHs).

**Extended Data Fig. 2:**
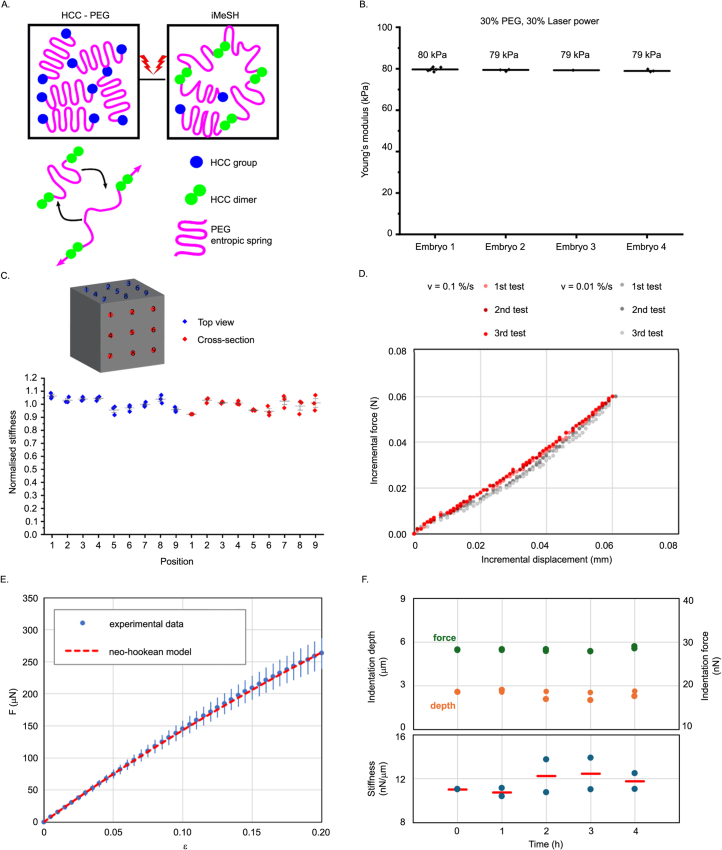
Validation of i3D bioprinted structure material properties. **a**. Schematic showing conversion of HCC-PEG polymer into an iMeSH structure through photocrosslinking using two-photon irradiation. Elastic PEG deformation caused by force application (magenta arrows) is schematically illustrated. **b**. AFM quantification of Young’s modulus of i3D shapes printed in four different embryos with equivalent polymerisation settings. The results have been derived from force-distance curves recorded at indentation depths of 3 μm and rates of 0.3 μm/s (Embryo 1,2,4) or 0.5 μm/s (Embryo 3). **c**. Repeated AFM measures on the top view and cut cross-section of a cube constructed by photo-crosslinking of 8 arm PEG B. The measured cube is shown schematically. Measurements were as indicated in the diagram, at 50 μm apart and repeated 3 times for each position. Thegraph represents normalised values of stiffness over the average stiffness of both top and cut cross-section. Points represent the 3 repeated measurements in each position, plotted also as mean value +/- standard deviation. Indentation was made with PARK pyramidal tip at 1um and velocity of 0.5 μm/sec. **d**. Incremental compressive force vs. displacement data for a sample of PEG hydrogel at repeated compressive loading at different strain rates (0.1 %/s and 0.01 %/s). Force and displacement increments are evaluated from the initial 5% pre-strain level. Three loading cycles are shown for each strain rate. The slope of the regression line corresponds to the stiffness of each sample. **e**. Stress-strain testing of i3D polymer and corresponding prediction derived from a neo-Hookean model. Points represent the mean +/- SEM, n = 8 i3D shapes. **f**. Indentation force, indentation depth, and stiffness vs. time from AFM indentation at constant imposed force on two PEG hydrogel samples. The stiffness values are calculated as ratio of indentation force and corresponding indentation depth. The almost constant values of indentation depth and stiffness vs. time (up to 4 hours), show the elastic behaviour of PEG hydrogel. Source data

**Extended Data Fig. 3:**
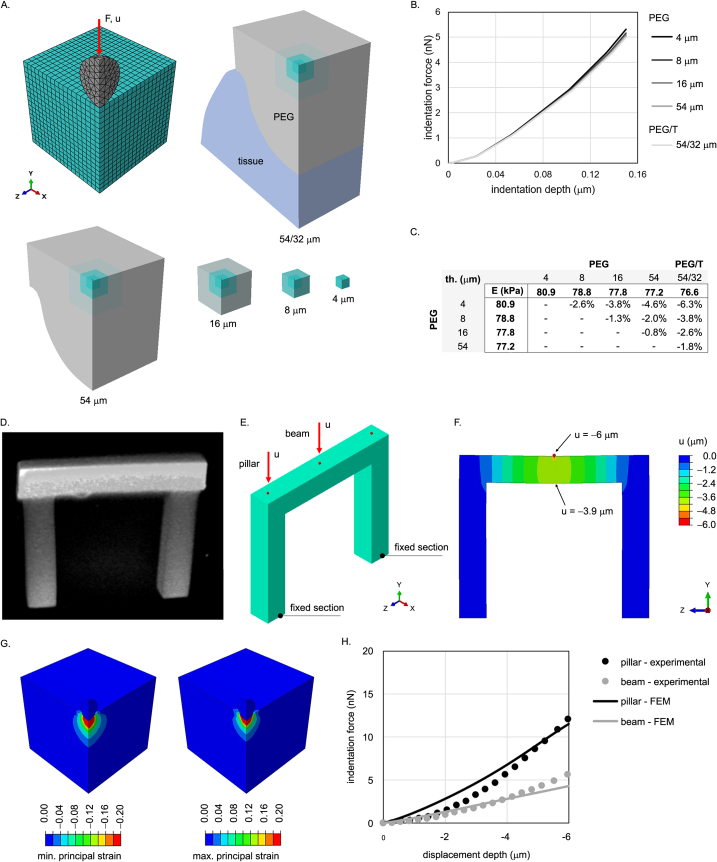
In silico analysis of AFM testing procedure and bending deformation of a bridge structure. **a**. Detail of FEM models in the contact region for a spherical indenter with diameter 0.8 μm. The mesh density in the contact region is the same for all the models. A double plane symmetry (X-Y and Z-Y) is considered. Geometry of the FEM models, assuming different thickness of PEG (4 μm, 8 μm, 16 μm, 54 μm), also in contact with embryonic tissue (PEG/T 54/32 μm), under the indentation region. Young’s modulus was set to 80 kPa and 8 kPa for PEG and embryonic tissue, respectively, with Poisson’s ratio of 0.49 for both materials. **b**. Indentation force vs. indentation depth for the different FEM models as estimated from the numerical analyses. **c**. Young’s modulus E estimated through a fitting procedure of the Hertz contact formula to the data of indentation force vs. indentation depth. At the intersection of each row and column, it is possible to get the percentage difference between the values of the Young’s modulus estimated for each pair of geometric configurations. These values are low, showing that the estimation of the Young’s modulus of PEG is marginally affected by the boundary conditions of the samples. **d**. Representative image of the bridge structure measured by AFM. 8 arm PEG A was photocrosslinked on functionalised PEG A glass slides to ensure attachment. **e**. FEM model geometry of the bridge sample tested with AFM indentation. Pillars and beam have a square transversal section with size 100 μm; the axis length is 550 μm and 600 μm for pillars and beam, respectively. The base of the pillars is fixed, to resemble the connection of the sample to the glass slide used in the experiments. The AFM indentation is performed by using a spherical indenter with diameter of 10.8 μm and cantilever stiffness of 2.9N/m. The indenter is applied on the top section of the pillars and on the middle section of the beam (separately). **f**. Colour map of the displacement (Y axis) for an indentation of 6 μm in the middle section of the beam. The displacement field shows that the beam also undergoes a bending deformation. **g**. Colour maps of maximum and minimum principal values of the nominal strain in the region of indentation for the case of indentation in the middle section of the beam. **h**. Indentation force vs. indentation depth measured by AFM experiment and estimated by FEM simulation. The data show a lower stiffness for the indentation in the middle section of the beam, because in this condition the indentation force is also affected by the bending deformation. Source data

A critical prerequisite for this application is the ability to fine-tune iMeSH material properties. Their stiffness (Young’s modulus) is related to the crosslinking of coumarin groups^23^, which also produces autofluorescence (Fig. 1e and Extended Data Fig. 2a). Force sensor material properties are typically inferred from measurements performed before implantation in vivo^13^. However, atomic force microscopy (AFM) can be performed directly on iMeSH structures printed in the chick neural tube, and an estimation of Young’s modulus can be obtained while taking into account the stiffness of surrounding tissues using computational mechanical modelling (Extended Data Fig. 3a–c). Young’s modulus can be reproducibly adapted to suit experimental needs either by adjusting the laser powers applied or by changing the concentration of the polymer used (Fig. 1f,g). Repeated measurements within the same embryos, and measurements comparing embryos, demonstrate high reproducibility (Extended Data Fig. 2b). Repeated measurements along the top of an iMeSH block and along a cut side demonstrate highly homogeneous stiffness (Extended Data Fig. 2c). Repeated cycles of compressive testing at different loading rates shows that iMeSH stiffness is independent of the force application rate (Extended Data Fig. 2d). Mechanical testing of the polymer demonstrates that it follows a linear stress–strain profile, which can be approximated by a neo-Hookean model (Extended Data Fig. 2e). Prolonged and repeated AFM measurements show purely elastic hydrogel behaviour for a time period of at least 4 h (Extended Data Fig. 2f).

## Inferring morphogenetic mechanics from iMeSH deformation

**Fig. 2:**
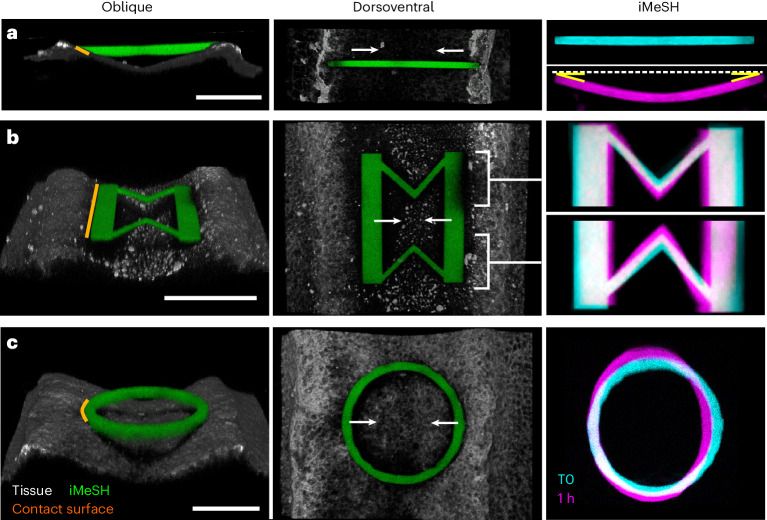
Optimization of force sensor shapes to quantify morphogenetic forces. **a**–**c**, Oblique and dorsoventral 3D reconstructions of a horizontal bar (**a**), a double V-shaped spring (**b**) and a cylinder (**c**), iMeSH shapes printed between chick embryo neural folds. Overlaid initial (T0) and deformed (at 1 h) geometries are shown. Orange lines show tissue contacts along which force is applied; white arrows indicate the direction of neural fold apposition; and yellow angles indicate highly symmetrical deformation. Scale bars, 100 µm.

**Extended Data Fig. 4:**
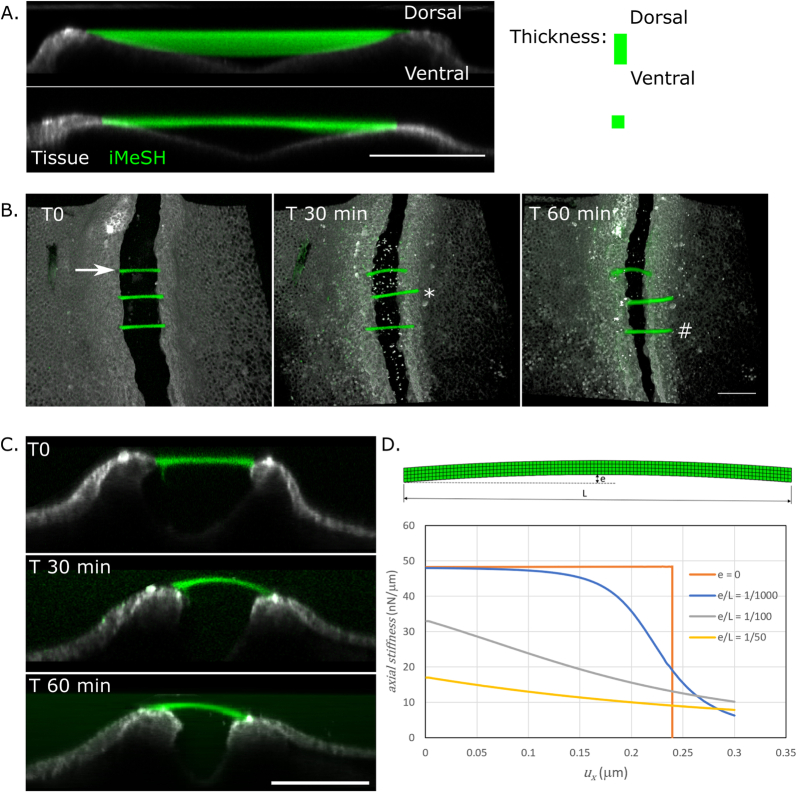
Horizontal bar force sensors. **a**. Horizontal bars printed superficially or to greater ventral depth between the neural folds of different embryos, showing ability to generate structures suspended between embryonic tissues.Images are representative of more than 3 replicates of thin and thick structures. **b**. Time series showing variable deformation of three bars between the neural folds of the same embryo. Arrow: shown in (C). *: quickly detaches. #: initially deforms, then detaches. **c**. Optical re-slice of the bar indicated by an arrow in (B) showing homogenous dorsal bending. **d**. Idealised FEM model showing highly non-linear force-displacement relationship between bars with slight differences in initial curvature. Perfectly straight bars (e = 0) resist very high axial force before deforming suddenly. Scale bars = 100 μm throughout. Source data

The iMeSH structures incorporated in the neuroepithelium become mediolaterally compressed within the closing lumen (Fig. 2). We assayed various potential iMeSH geometries to quantify forces from shape deformation. First, the custom finite element method (FEM) model of a bridge shape built on a glass substrate with a defined Young’s modulus was used to show that the force–deformation response of specific shapes can be predicted (Extended Data Fig. 3d–h). Simple iMeSH bars can be photo-printed to different depths between the neural folds (Extended Data Fig. 4a) and multiple shapes can be printed within an embryo (Extended Data Fig. 4b). These can deform as the neural folds come together, producing very even curvature, testament to the homogeneity of the iMeSH material (Fig. 2a). Two properties make this shape unsuitable for force quantification. The contact surface between the iMeSH and the neural fold is very narrow, producing a stress peak that commonly causes the bars to detach from the tissue (Extended Data Fig. 4b,c). Additionally, the shape’s stress–strain relationship is entirely nonlinear such that deformation resulting from forces applied to it depends entirely on its initial curvature or the direction of force application (Extended Data Fig. 4d).

**Extended Data Fig. 5:**
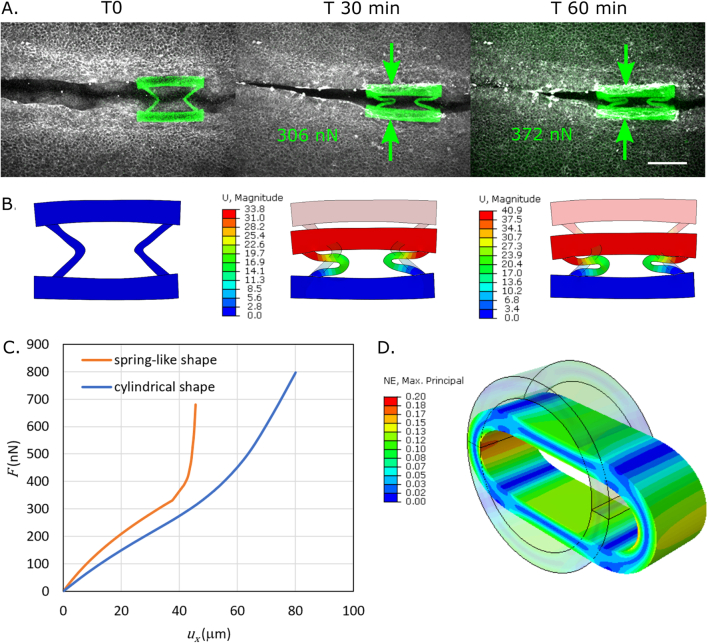
‘V’ spring force sensors. **a**. Confocal time series showing progressive deformation of a double ‘V’ iMeSH between chick neural folds. Green arrows indicate the direction of force application. Force values are those calculated from an individualised FEM models in (B). Scale bar = 100 μm. **b**. Individualised FEM models for the iMeSH shape in (A) showing agreement between empirically observed and modelled shape deformation. **c**. Force-displacement modelling of a double ‘V’ spring shape contrasted with a simple cylinder shape. Note the highly non-linear relationship of the ‘V’ springs compared with a cylinder shape. **d**. Visualisation of extreme deformation of an idealised iMeSH cylinder, exceeding deformations observed in vivo, compressed up to a 46% reduction of the initial diameter. This deformed configuration corresponds to a maximum local strain of 20%. Ad-hoc FEM models are required for higher deformation ranges. Source data

A second shape was developed to increase the predictability of the deformation and force application: stiff bars along the neural folds interconnected by two V-shaped springs (Fig. 2b). This shape can be reproducibly printed in vivo and produces a medial narrowing of the V springs when compressed (Fig. 2b and Extended Data Fig. 5a). Custom FEM modelling of individual shapes shows that the neural folds generate forces in the low hundred nano-newtons (Extended Data Fig. 5b,c). However, this shape’s deformation–force relationship is highly nonlinear at biologically relevant deformation magnitudes (Extended Data Fig. 5c). At small deformations, individualized FEM models show that 77.9 ± 39.1 nN (mean ± standard deviation, *n* = 4; tissue contact surface, 170 µm long) is required for the neural tube to compress the iMeSH’s V shapes by 10% of their initial width (8 µm).

**Fig. 3:**
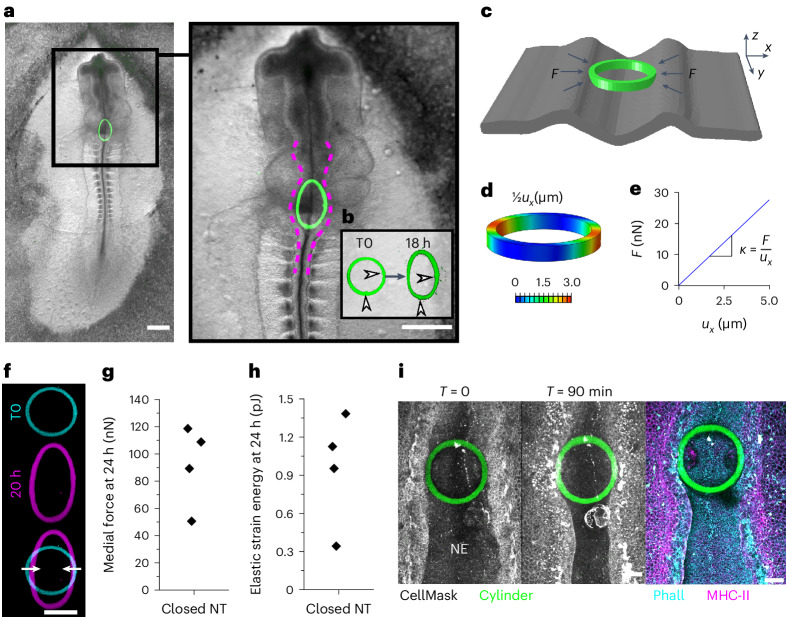
Quantification of medial force applied by the closed neural tube. **a**, Bright-field view of a chick embryo 18 h after an iMeSH cylinder (green) was bioprinted within its open neural tube. Dashed lines indicate the neural folds. Scale bars, 500 µm. **b**, Confocal 3D reconstructions of the cylinder in the same embryo following bioprinting (T0) and 18 h later. Arrowheads indicate small landmarks incorporated in the cylinder, demonstrating minimal rotation. **c**, FEM model of an iMeSH cylinder and surrounding tissue based on 3D reconstruction of the specific morphometry, with the representation of contact forces (*F*) between tissue and cylinder. In the reference system, *x* is the medial–lateral direction, *y* the craniocaudal direction and *z* the dorsoventral direction. **d**, Contours of absolute displacement in the mediolateral direction (*u*_*x*_). **e**, Resultant contact force versus narrowing in the mediolateral direction. The slope of the curve corresponds to the cylinder structural stiffness, *k*. **f**, Projected image of a cylinder printed in a chicken rhombocervical neuropore immediately after printing and in a deformed state within the lumen of the neural tube 20 h later. Scale bar, 100 µm. **g**,**h**, Quantification of medial force applied (**g**) and elastic energy stored within compressed cylinders (**h**) incorporated in a partially closed neural tube (NT), 24 h after printing. Points represent individual embryos. **i**, Representative embryo immediately after iMeSH printing and 90 minutes later to visualise the iMeSH cylinder. The same embryo was fixed and stained with the plasma membrane dye CellMask, phalloidin (Phall) to label F-actin, and immuno-labelled to detect myosin heavy chain (MHC)-II. NE, neuroepithelium; T, time; scale bars, 50 µm. Source data

**Extended Data Fig. 6:**
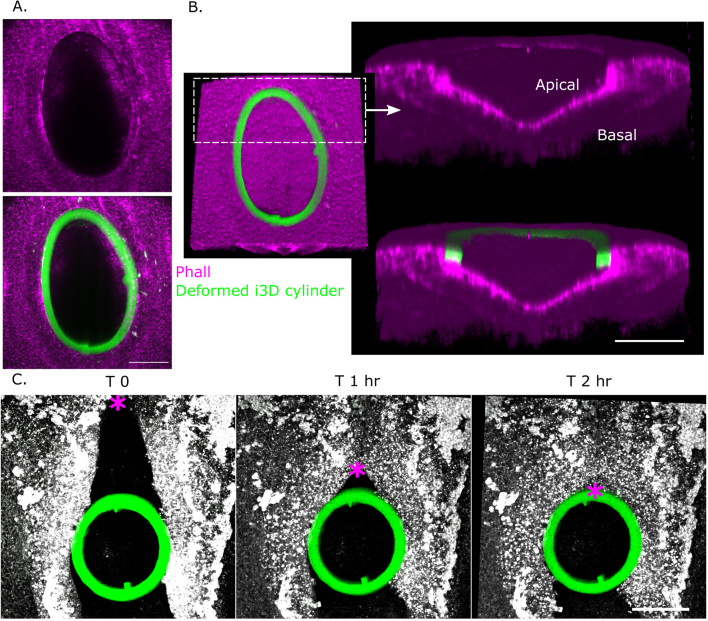
i3D cylinder incorporation within the closing neural tube. **a, b**. Representative embryo (of 4 independent embryos cultured overnight) showing encircling of an i3D cylinder by the surface ectoderm and neural folds, leaving an open defect, shown as maximum projections (A) and 3D confocal reconstructions (B). Note the persistent apical enrichment of phalloidin-labelled neuroepithelial F-actin. Scale bar = 100 μm. **c**. Live-imaging of a CellMask-labelled embryos (representative of 6 independent embryos with stiff cylinders) showing progression of rostral-to-caudal zippering (*) until it encircles a stiff cylinder incorporated within its neural folds. Note triangle and square shapes incorporated in the cylinder illustrating control over printed geometries and lack of rotation. Scale bar = 100 μm. Source data

By contrast, simple cylinder shapes provide a quasilinear deformation–force relationship over the relevant range of deformations (Fig. 2c and Extended Data Fig. 5c,d). Cylinder shapes incorporated between the neural folds stop the closure of the adjacent neural tube, forming a localized open defect (Fig. 3a,b and Extended Data Fig. 6a,b). Over long timescales, fusion of the flanking zippering points progresses to encircle the printed object (Extended Data Fig. 6a,b), empirically dissociating local mediolateral apposition from rostrocaudal zippering. Cylinders with excessive structural stiffness also prevent neural fold apposition as the zipper advances to contact the iMeSH, but do not show any deformation from which forces can be quantified (Extended Data Fig. 6c). It is therefore necessary to tune the structure of the iMeSH force sensor to match the tissue force-generating properties.

**Extended Data Fig. 7:**
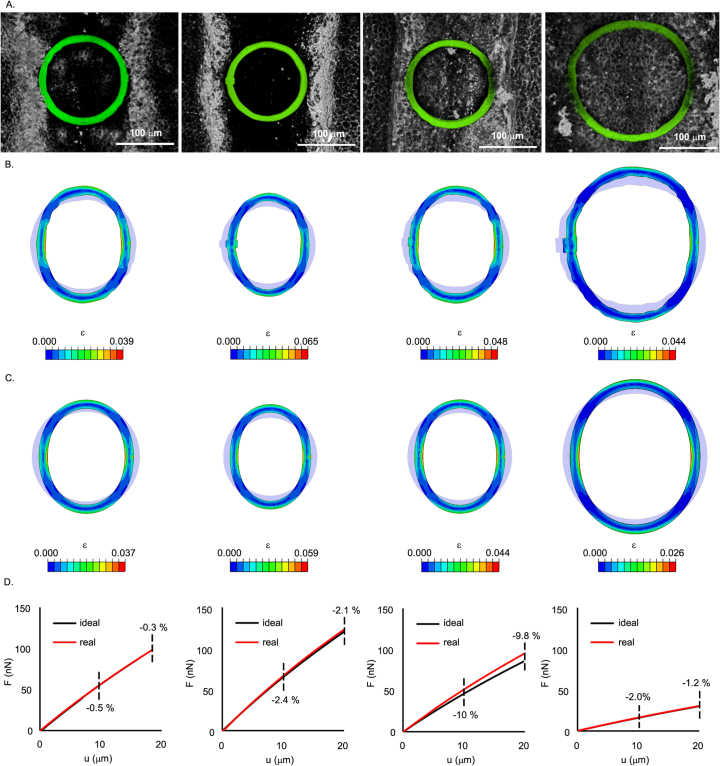
Effect of geometrical inhomogeneities on contact force quantification. **a**. Four examples, from independent embryos, of iMeSH cylinder with different levels of irregularity in the wall thickness and diameter. Scale bar = 100 μm. **b**. Contours of the logarithmic strain in the medial-lateral direction of specific iMeSH cylinders with real geometry, featuring inhomogeneities in the wall thickness and diameter. **c**. Contours of the logarithmic strain in the medial-lateral direction of idealised iMeSH cylinders, with constant wall thickness and diameter, corresponding to the mean value of the realNones. **d**. Comparison of force-displacement curves of real and idealized iMeSH cylinders. TheNpercentage errors due to modelling of an idealized geometry with respect to real one are reported at two values (10 μm and 20 μm) of the displacement in the medial-lateral direction. Source data

**Extended Data Fig. 8:**
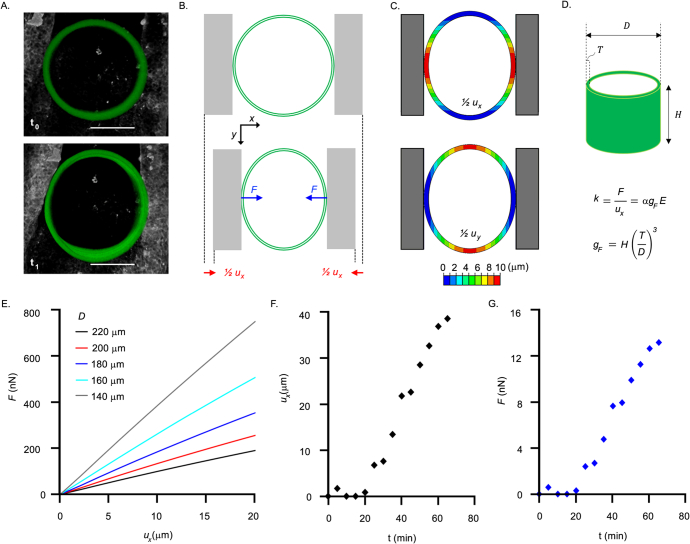
Development of Finite Elements Method (FEM) models and derivation of a parametrized equation to evaluate force from cylinder iMeSH shapes. **a**. Dorsoventral view of an i3D cylinder incorporated within the neural folds of a representative chick embryo at different time points (t0 = 0, t1 = 65 min). Scale bar = 100 μm. **b**. Scheme showing the compressive force F on the cylinder walls, due to the growth and folding of the neural tube, and the corresponding deformation of the cylinder. In the reference system, x is the medial-lateral direction and y the cranio-caudal direction. **c**. Contours of absolute displacement in the medial-lateral direction (ux) and in the cranio-caudal direction (uy). The cylinder narrowing in the medial-lateral direction from experimental data is the input of numerical analysis, which allows to estimate the corresponding contact force. **d**. The cylinder stiffness k, as the ratio between the contact force F and the displacement ux, is proportional to the hydrogel Young’s modulus E and to a geometric factor gF, which depends on cylinder height H, diameter D and wall thickness T. The scalar α in the parametric equation is a fitting constant (α = 7.718). **e**. Contact force (F) versus displacement (ux) for cylinders with the same height (H = 100 mm), thickness (T = 25 mm) and elastic modulus (E = 80 kPa) and varying diameter D, from numerical analyses. **f**. Displacement (ux) versus time (t) measured in a i3D cylinder incorporated in a chick embryo at different time points in a representative experiment. **g**. Contact force values versus time estimated via the parametric equation using experimental displacement data as input. Source data

Force quantification from structure-specific FEM models is closely approximated by an idealized cylinder shape (Extended Data Fig. 7). Using FEM, we derived a parameterized equation whereby iMeSH cylinder deformation serves as a generalizable read-out of the force applied (Fig. 3c–e and Extended Data Fig. 8). Modelled cylinder narrowing and contralateral elastic expansion are quasilinearly related to the mechanical force applied by lateral contacts (Fig. 3e and Extended Data Fig. 5c). The linear relationship is reliable up to approximately 20% strain (defined as the percentage change in cylinder width), although a shape-specific FEM can be used to quantify forces from larger deformations. The medial compression and perpendicular elongation of iMeSH cylinders predicted in silico is observed in vivo (Fig. 3f).

At late developmental time points, after the completion of rhombocervical neuropore closure, the neural tube continues to compress the iMeSH force sensor. The cylinder’s mediolateral narrowing relative to its original width allows the calculation of force (Fig. 3g). Force values obtained at late time points should not be considered absolute given the high deformation magnitudes and evolution of contact points around the cylinder’s circumference. However, the polymer material properties are very stable over time: iMeSH with an AFM-calculated stiffness of 80.56 ± 0.14 kPa after printing retains a stiffness of 80.58 ± 0.35 kPa 15 days later. FEM models of cylinder shapes show that a medial compression of 8 µm requires the neural tube to apply 54.98 ± 11.20 nN (mean ± standard deviation, *n* = 4; tissue contact surface, ~75 µm long). The quantification of force applied per unit length is therefore consistent between the two shapes modelled. The force applied to the iMeSH cylinder is stored as elastic energy in the low pico-joule range (Fig. 3h). Converting this mechanical energy value into biological currency, 1 pJ can be stored in approximately 1 × 10^7^ ATP molecules^25^, although the biological conversion of chemical to mechanical energy is likely to be highly inefficient.

**Extended Data Fig. 9:**
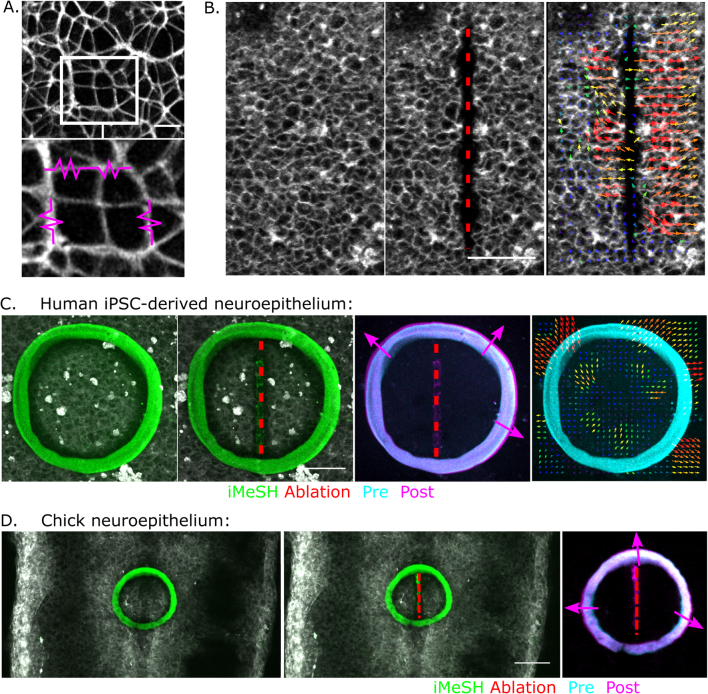
iMeSH cylinders can be directly attached to tissues. **a**. Apical surface of an iPSC-derived neuroepithelial cell layer showing cortical F-actin expected to be under tension (magenta springs). Scale bar = 10 μm. **b**. Laser ablation of a vertical line in the apical surface of a live-imaged iPSC-derived neuroepithelium. Arrows indicate displacement visualised by PIV. Scale bar = 50 μm. **c, d**. iMeSH cylinders were printed attached to the apical surface of iPSC-derived neuroepithelial cells (C) or chick embryos (representative of 7 embryos with laser ablations with printed cylinders) (D). A straight laser ablation (dashed red line) is created in the neuroepithelium and the iMeSH re-imaged immediately after. Merged and PIV visualisation illustrate cylinder expansion, absorbing elastic energy released from the ablation site. Images are representative of 6 independent wells. Green fluorescence along the ablation line is present even without i3D polymer and likely reflects a change in laser-ablated CellMaskTM used to visualise cell borders. Scale bars = 50 μm. Source data

We hypothesize that this energy derives from the persistent actomyosin-dependent contractility of cells (Fig. 3i and Extended Data Fig. 9a). Release of the contractile energy is commonly visualized by laser ablation (Extended Data Fig. 9b). The iMeSH structures can be printed attached to neuroepithelial cells’ apical surface, both in cells generated from human induced pluripotent stem cells (iPSCs; Extended Data Fig. 9c) and the chick embryonic neuroepithelium in vivo (Extended Data Fig. 9d). Ablation of cells within the iMeSH shape releases the tension in their cell borders, deforming the iMeSH, demonstrating this material’s ability to be crosslinked and attached to cell layers (Extended Data Fig. 9c,d). Neuroepithelial apical constriction is well-established to be an essential source of mechanical force narrowing the neuropores^10,26,27^.

## Pro-closure versus anti-closure force balance in neurulation

**Fig. 4:**
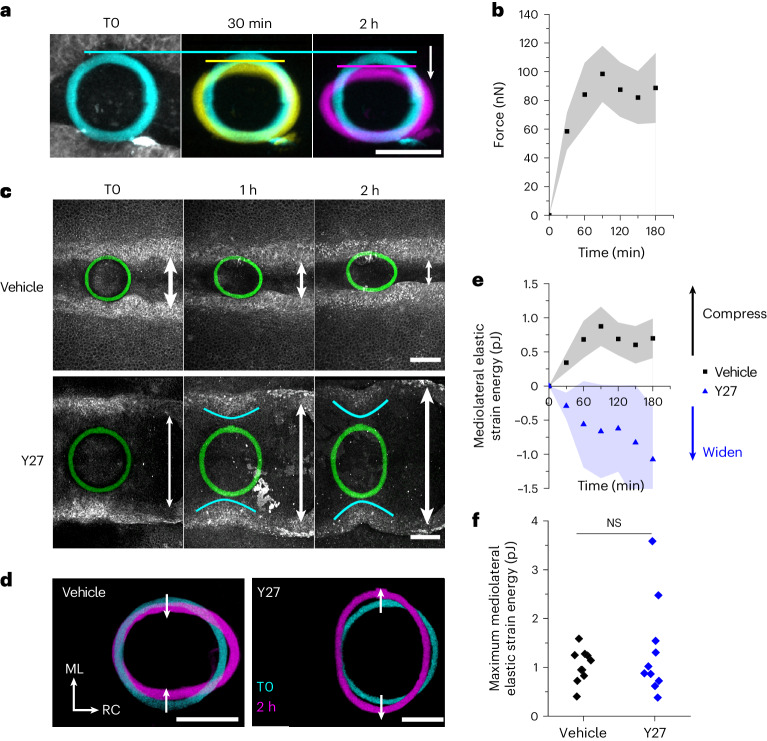
Dynamic quantification of morphogenetic mechanics during neurulation. **a**, Illustrative iMeSH cylinder showing progressive medial displacement (arrow). The horizontal lines indicate the top of the cylinder. Scale bar, 50 µm. **b**, Dynamic profiling of medial compressive strain experienced by the iMeSH cylinder. Points represent the mean ± 95% confidence interval (CI), *n* = 10 vehicle-treated embryos. **c**, Sequential images at indicated time points in a vehicle-treated embryo and one treated with 20 µM of the ROCK inhibitor Y27632 (Y27). White arrows indicate the width of the neuropore. Cyan lines illustrate bending of the neural folds as they pull on the attached iMeSH cylinder. Scale bars, 100 µm. **d**, Merged reconstructions of the iMeSH cylinders in **a** at two time points. ML, mediolateral; RC, rostrocaudal. Scale bars, 100 µm. **e**, Dynamic profiling of mechanical force applied to the iMeSH cylinder. Points represent the mean ± 95% CI, *n* = 10 per group; vehicle embryos are those force-profiled in **b**. **f**, Maximum potential energy imparted by each embryo during live imaging. Points represent independent embryos. NS, not significant. Two-tailed *t*-test, *P* = 0.375. Source data

**Extended Data Fig. 10:**
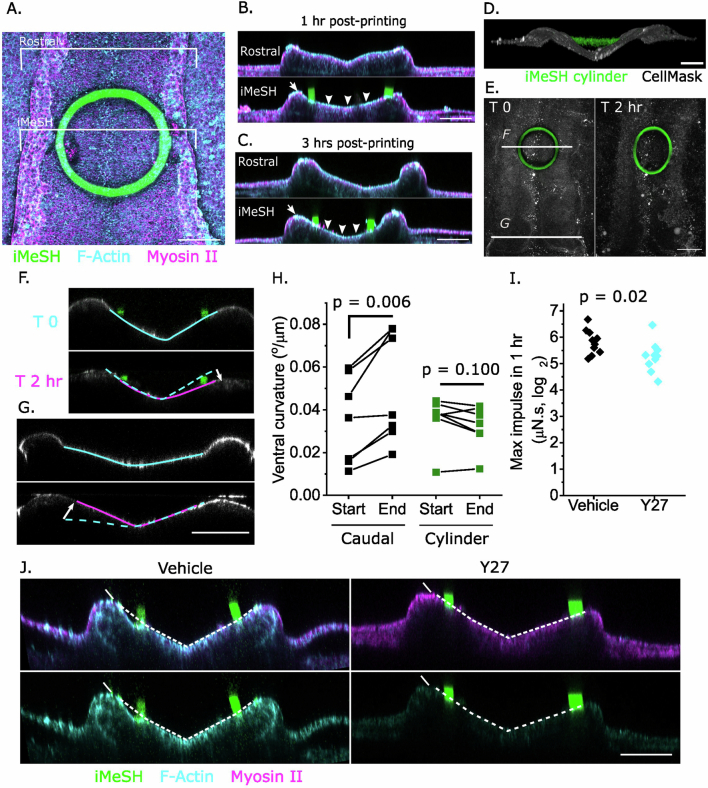
iMeSH cylinders do not disrupt tissue morphology and allow to quantify actomyosin-dependent pro-closure impulse. **a**. Dorsoventral confocal image showing F-actin and myosin-II wholemount staining of a chick embryo with an iMeSH cylinder between the neural folds. Note this also illustrates the robust attachment of iMeSH to tissues, withstanding washes in solution and repositioning for imaging. Brackets through the iMeSH and 100 μm rostral to it indicate the positions of optical crosssections in B-C. **b, c**. Optical cross-sections through actomyosin-stained embryos fixed 1 hour or 3 hours following iMeSH printing. Arrowheads indicate persistent apical F-actin enrichment, arrows indicate normal morphology of the neural fold tips lateral to the iMeSH. In A-C: images are representative of 3 independent embryos. Scale bars = 100 μm. **d**. 3D reconstruction showing an iMeSH cylinder suspended between the RNP neural folds. Scale bar = 100 μm. **e**. Projections showing cylinder deformation over two hours. F and G indicate the positions of the corresponding panels. Scale bar = 100 μm. **f**, **g**. Optically-resliced projections showing the curvature of the ventral neuroepithelium (annotated) below the neural fold tips. Dashed lines indicate the T0 apical contour, and the white arrows indicate the change in bending. Scale bar = 50 μm. **h**. Quantification of the ventral neuroepithelial curvature at the start of imaging and 1.5-2 hours later in seven independent embryos caudal to, or at the level of, the cylinder. P values by twotailed T-test paired by embryo. **i**. Impulse was calculated as the maximum force applied to the i3D cylinder within 1 hour. Points represent independent embryos. Log2-normalised values of vehicle embryos are compared against Y27-treated embryos by t two-tailed test, n = 10 per group. **j**. Optical cross-sections through the neuroepithelium of wholemount stained vehicle and ROCKinhibited embryos with iMeSH cylinders showing diminished F-actin in the latter. Scale bar = 50 μm. Images are representative of 3 independent embryos per treatment group. Source data

The combination of iMeSH force sensor bioprinting with time-lapse imaging makes it possible to dynamically profile morphogenetic forces in vivo (Fig. 4a,b). To quantify force, iMeSH cylinders were attached suspended between the neural folds. Neuroepithelial cells retain their expected F-actin enrichment relative to surrounding tissues in embryos with iMeSH cylinders between their neural folds (Fig. 3i and Extended Data Fig. 10). No abnormal compression, actomyosin disruption or accumulation of tissues along the iMeSH shapes is observed (Extended Data Fig. 10b,c). We would expect cells compressing the hydrogel to be exposed to different stiffnesses and mechanical forces, compared to the ones they normally experience. Mechanical deformation of cells would be expected to change the tension or compression, which are known to trigger signalling cascades including piezo channel opening^28^ and YAP nuclear localization^29^. For example, we recently reported that YAP nuclear levels in the surface ectoderm of mouse embryos are related to local cell border tension^30^. Local mechanotransduction events triggered by contact with the hydrogel material, which is stiffer than the natural environment the cells encounter, could result in different cell responses. This has been shown, for example in the context of foreign body reactions to medical implants^31–33^. Mechanotransduction may lead to transcriptional changes, the release of inflammatory mediators or other changes in the tissue. However, these are unlikely to alter the interpretation of the methods we describe as the timescales required for these changes are different from those of the force measurements reported.

The apical neuroepithelium curves dorsally, and iMeSH structures suspended between the neural folds resist this deformation only locally (Extended Data Fig. 10d–h). Medial apposition of the neural folds applies an incremental compressive force to the iMeSH rim, causing it to displace medially (Fig. 4a). The iMeSH cylinder strain, defined as the percentage change in width, increases progressively as the embryo applies compressive forces, up to approximately 100 nN within a developmentally relevant window of one to two hours, before reaching a plateau or tending to decrease (Fig. 4b–e).

Pharmacologically inhibiting the myosin-activating kinase ROCK is known to stop neural fold elevation in chick and mammalian embryos^26,27^. We observed the loss of F-actin (Extended Data Fig. 10j), as previously reported^26^, and a progressive widening of the neural folds in ROCK-inhibited embryos (Fig. 4c). This presents an additional force quantification challenge: force sensors, such as cantilevers, simply placed between the neural folds deform only when the surrounding tissue compresses them. Anchoring iMeSH cylinders directly to embryonic tissue solves this problem, allowing them to be stretched by tissue expansion (Fig. 4c–e). ROCK-inhibited embryos generate less force per unit time than vehicle controls: their maximum impulse within 60 min is significantly lower (Extended Data Fig. 10i). Nonetheless, it is remarkable that the absolute anti-closure energy imparted by ROCK-inhibited embryos is comparable to the pro-closure equivalent in controls (Fig. 4f). Potential force-generating mechanisms not interrupted by a blockade of Rho/ROCK activation include ongoing cell proliferation^27^, hydrostatic extracellular matrix expansion^34^ and cell migration^35^.

Thus, iMeSH force sensors printed with high spatial resolution and positional accuracy, combined with time-lapse live imaging, enable the quantification of the mechanical energy generated during vertebrate neural tube closure. The iMeSH quantifies the nano-newton forces generated by embryonic tissues with no need for specialist equipment beyond readily available two-photon microscopes and the liquid polymer. This technology is highly versatile, readily accommodating differences in the initial morphology and direction of force generation, allowing the dynamic profiling of both compressive and stretching forces. Photo-printing a simple cylinder shape allows forces to be calculated from deformation using a generalized equation. Other methods can be used to quantify pico-newton forces^13^, pressure^16^, molecular strain^12^ or deflection^21^, but iMeSH is unique in its ability to quantify forces applied in unpredictable directions with variable tissue geometries at high force resolution. The application of this technology has already provided unexpected insights into the delicate balance between pro- and anti-morphogenetic forces that, when disrupted, may produce severe birth defects.

## Methods

### Chick embryo culture and inhibitor treatment

Studies were performed under the regulation of the UK Animals (Scientific Procedures) Act 1986 and the National Centre for the 3Rs’ Responsibility in the Use of Animals for Medical Research (2019). Fertile Dekalb white eggs (Henry Stewart) of species *Gallus gallus* were incubated at 37 °C for 34 h to reach Hamburger and Hamilton (HH) stage 8. Embryos were dissected and put in Early Chick (EC) culture at embryonic stages HH 8–11 following a published protocol^36^. Excess yolk was washed off using Pannett–Compton saline. Embryos with rhombocervical or posterior neuropores were selected. The vitelline membrane was windowed using a tungsten needle to expose the neuropore. For inhibitor studies, ROCK inhibitor (Y27632; Cell Guidance Systems) was reconstituted with phosphate buffered saline (PBS) at a stock concentration of 10 mM. The inhibitor was mixed with agar–albumen for the preparation of EC culture plates at a final concentration of 10 µM (*n* = 3) or 20 µM (*n* = 7): no differences were observed between these concentrations, so they were combined for all analyses. The *n* numbers used for *P* values are shown in the corresponding images. For live imaging, the embryos were transferred to the inhibitor plates just prior to vitelline membrane windowing and were exposed to the inhibitor for 20 min before the start of imaging. Sex cannot be visibly determined at the embryonic stages used and the embryos were not genotyped for sex.

### iPSC culture and neuroepithelial differentiation

The iPSC line HO-193b (ref. ^37^) was differentiated into neuroepithelial cells over eight days using dual-SMAD inhibition^38^ as previously reported^39^. The HO-193b line was generated from human amniocytes. The reprogramming to human iPSCs was performed by using a previously developed messenger RNA (mRNA)-mediated microfluidic strategy in the Elvassore group. No authentication procedure was followed for the HO-193b line. The cell line used in this study was tested monthly for mycoplasma contamination, and it tested mycoplasma negative. No cell lines used in this study are present in the International Cell Line Authentication Committee (ICLAC) register.

### Bioprinting, time-lapse live imaging and laser ablation

After vitelline membrane windowing, embryos were stained with 1:100 CellMask deep red plasma membrane (C10046, Invitrogen, Paisley) in PBS for 15 min at 37 °C. Excess CellMask was washed off with PBS. For inhibitor studies, Y27632 was diluted at 20 µM with CellMask and PBS for staining and washing, respectively. Then 3 µl of 30% 7-hydroxycoumarin-3-carboxylic acid (HCC) eight-arm PEG in phosphate buffered saline without calcium and magnesium (DPBS) were added on the embryo over the neuropore. The embryo was then moved to the heated stage (37 °C) of a Zeiss Examiner LSM 880 confocal microscope for bioprinting. Cylinders were drawn as regions of interest (ROIs)in ZEN 2.3 software, overlapping with the neural folds for attachment. Two-photon hydrogel crosslinking was performed using a ×20, numerical aperture 0.7, EpiPlan Apochromat dry objective (working distance, 1.3 mm). Printing was performed using a Mai Tai laser (SpectraPhysics Mai Tai eHP DeepSee multiphoton laser, 1,436.1 mW maximum power) at 700 nm and 30% laser power. The *x* and *y* pixels were 0.25 µm; standard pixel dwell time, 0.47 µs; *z* step, 0.7 µm; and averaging, 4. Live imaging was performed using the same objective with *x* and *y* pixels of 0.59 µm and a *z* step of 1 µm (pixel dwell time, 0.77 µs; speed, 8; averaging, 1; bidirectional imaging, 1,024 × 1,024 pixels). Imaging lasers used were 488 nm at 1% and 633 nm at 0.50% laser power in order to obtain morphologically accurate information with minimal phototoxicity. The time step was 30 min.

Laser ablation was performed with the same Mai Tai laser as previously described^40^.

### Immunostaining

Images are representative of observations in five independent embryos. The primary antibody against myosin IIb (CMII 23s) was purchased from Developmental Studies Hybridoma Bank at a stock concentration of 44 µg ml^–1^. Embryos were fixed with 4% paraformaldehyde (PFA) overnight at 4 °C. They were permeabilized with 0.1% Triton X-100 in PBS (PBT) for 1 h at room temperature, blocked overnight in 5% BSA/PBT at 4 °C (BSA, bovine serum albumin) and incubated in a 1:10 dilution of primary antibody in blocking solution. After three 30 min washes in blocking solution at room temperature, the embryos were incubated in a 1:500 dilution of Alexa Fluor 568 conjugated secondary antibody (Life Technologies) and 1:200 Phalloidin 647 (Invitrogen), both in blocking solution. Excess secondary antibody was removed by washing with PBT at room temperature. For imaging, stained embryos were held in place with tungsten needles on 4% agar dishes and imaged in PBS on a Zeiss LSM 880 confocal microscope. The objective was a dipping ×10, numerical aperture 0.5, Plan Apochromat with *x* and *y* pixel sizes of 0.83 µm and a *z* step of 2.78 µm (pixel dwell time, 0.77 µs; speed, 8; averaging, 2; bidirectional imaging, 1,024 × 1,024 pixels). Images were processed with ZEN 2.3 software and visualized as a maximum or 3D projections in Fiji^41^.

### Image analysis and force quantification

Acquired sequences were registered using the Fiji plug-ins Correct 3D Drift and StackReg. Cylinder deformation was measured in Fiji using a bounding rectangle. Following registration, the zippering rate was calculated using the Fiji Manual Tracking plug-in and the Chemotaxis Tool (Ibidi). When needed, images were denoised using PureDenoise^42^ in Fiji. Cylinder dimensions were measured in a confocal image acquired immediately after i3D printing using line tools in Fiji. In order to calculate the cylinder’s width, maximum projections of all time points were registered using rigid body registration with StackReg in Fiji, the cylinder was manually outlined and its mediolateral width was measured using the bounding rectangle tool. Force was calculated from the mediolateral deformation of the cylinder using a parametrized equation (Extended Data Fig. 8). Work, which results in potential energy being stored within the force sensor, was calculated as the area under the force versus displacement curve. Impulse was calculated as force generated within each 60 min period, multiplied by time.

Particle image velocimetry was performed in Fiji^43^.

### Stress–strain testing and AFM-based force spectroscopy

The stress–strain mechanical behaviour of eight-arm PEG hydrogel was analysed through uniaxial tensile tests by means of a Bose ElectroForce Planar Biaxial Test Bench instrument (TA Instruments) with a load cell of 22 N, at a strain rate of 0.1% s^−1^, on eight samples. Testing was performed at up to 20% of nominal strain.

Compressive mechanical behaviour of the eight-arm PEG hydrogel was analysed through unconfined compression tests using a Bose ElectroForce Planar Biaxial Test Bench instrument (TA Instruments) with a load cell of 22 N, at two strain rates—0.1% s^−1^ and 0.01% s^−1^—on five samples. Testing was performed from 5% to 10% of nominal compressive strain by repeated loading ramps. Samples were completely immersed in PBS for the duration of the tests.

AFM measurements were conducted using an XE Bio AFM instrument (Park Systems). The force–displacement curves were acquired using PPP-CONTSCR-10 pyramidal tips mounted on Si_3_N_4_ cantilevers with a nominal spring constant of 0.2 N m^–1^ (NanoSensors). Cantilever spring constants were calibrated by the manufacturer prior to use. The sensitivity of each cantilever was adjusted by measuring the slope of the force–distance curve acquired on a hard reference material prior to each experiment. Indentation experiments were repeated at least three times for each sample, at different locations. All AFM measurements were done in a fluid environment (PBS) at room temperature. The Young’s modulus was calculated by applying a fit of the Hertz model to the force–distance curve, assuming a Poisson ratio of 0.5, as is common practice for PEG hydrogels^44^. Preliminary in silico analyses of the AFM testing procedure were carried out to evaluate the effects of boundary conditions on the estimation of Young’s modulus (Extended Data Fig. 3).

### In silico analysis of the neural-tube/iMeSH interaction

FEM-based numerical models were developed by means of Abaqus/CAE and Abaqus/Standard (SIMULIA, Dassault Systèmes). FEM models of iMeSH cylinders and neural tube tissue were obtained from the 3D geometry of representative experiments. The mechanical behaviour of hydrogel and tissue were described with an isotropic, hyper-elastic, almost incompressible neo‐Hookean model, included in Abaqus/Standard. The constitutive parameters were set to correspond to Young’s modulus *E* = 80 kPa and 25 kPa for the hydrogel and the tissue, respectively. The values of Young’s modulus were measured through AFM indentation. Cylinder and tissue solid regions were meshed with hexahedral and tetrahedral elements, respectively, both with hybrid formulations to avoid numerical instabilities due to the almost incompressible behaviour. Nonlinear static analysis was carried out, simulating the progressive shifting of tissue folds and the corresponding deformation of the cylinder, up to the displacement values measured experimentally on the cylinder diameter in the medial–lateral direction. Contact forces between tissue and cylinder surfaces were computed from numerical results as an index of the closing capability of tissue.

### Estimation of iMeSH nominal stiffness

Custom FEM models were developed for V spring shapes based on experimentally determined geometries. Idealized bar FEM models were used to illustrate the effect of initial curvature on the displacement–force relationship.

FEM models with a simplified geometry were developed to evaluate the structural stiffness of the iMeSH cylinder when varying different parameters. In detail, several models were considered, varying cylinder height *H* between 20 µm and 100 µm; diameter *D* between 140 µm and 220 µm; wall thickness *t* between 5 µm and 25 µm; and hydrogel Young’s modulus *E* between 5 kPa to 80 kPa. Neural tissue folds were modelled as two rigid surfaces that got progressively closer in the medial–lateral direction. The contact force between the cylinder and rigid surfaces was computed from numerical analyses of each different condition, and the corresponding cylinder stiffness *k* was obtained as the ratio between the contact force and medial–lateral displacement. In the case of small displacements, the stiffness *k* can be approximated as a constant and obtained through a parametric equation of the type

$$k=\alpha \times H\times {\left(\frac{t}{D}\right)}^{3}\times E$$

where *α* is a fitting constant to be determined from the overall set of numerical results by means of an optimization procedure based on the least-squares method. The optimization procedure was implemented in a user routine developed in the open-source software Scilab (v.6.1.0, Esi Group), obtaining *α* = 7.718. This parametric equation allows the evaluation of nominal stiffness of the cylinder depending on its size (height, diameter and thickness) and Young’s modulus, in the above-mentioned ranges for the different parameters. Once the displacement of the cylinder diameter in the medial–lateral direction is measured from an experiment, it is used as an input, and the contact force between tissue and cylinder can be estimated at each instant by multiplying by the stiffness *k*.

### Statistical analysis

For qualitative end-points, observations were made in at least three independent embryos. For quantitative end-points, individual embryos were the unit of measure. Statistical comparisons between two groups of normally distributed data and equal homogeneity of variance were by two-tailed *t*-test, and those for non-parametric data by the Mann-Whitney U test in Origin 2020.

### Reporting summary

Further information on research design is available in the Nature Portfolio Reporting Summary linked to this article.

## Online content

Any methods, additional references, Nature Portfolio reporting summaries, source data, extended data, supplementary information, acknowledgements, peer review information; details of author contributions and competing interests; and statements of data and code availability are available at 10.1038/s41563-024-01942-9.

## Supplementary information

Reporting Summary

## Source data

Source Data Figs. 1, 3 and 4 Statistical source data for Figs. 1, 3 and 4.

Source Data Extended Data Figs. 1–10 Statistical source data for Extended Data Figs. 1–10.

## Data Availability

The raw microscopy data supporting the findings are available via Zenodo at 10.5281/zenodo.10988529 (ref. ^45^). Additional data are available from the corresponding authors upon request. Source data are provided with this paper.
